# Mesenchymal stem cells, not conditioned medium, contribute to kidney repair after ischemia-reperfusion injury

**DOI:** 10.1186/scrt489

**Published:** 2014-08-21

**Authors:** Li Xing, Rui Cui, Lei Peng, Jing Ma, Xiao Chen, Ru-Juan Xie, Bing Li

**Affiliations:** Department of Nephrology, 2nd Affiliated Hospital of Harbin Medical University, 246 Xuefu Road, Nangang District, Harbin, 150086 People’s Republic of China; Department of Nephrology, 1st Affiliated Hospital of Harbin Medical University, 23 Youzheng Road, Nangang District, Harbin, 150001 People’s Republic of China

## Abstract

**Introduction:**

Studies have shown that stem cells exert their therapeutic effects on acute kidney injury (AKI) through paracrine/endocrine actions. If the protective effect is mediated in an endocrine manner, the injection of the factors that these cells secrete could be effective, but the effect of conditioned medium (CM) remains controversial.

**Methods:**

In this study, we cultured mesenchymal stem cells (MSCs) and then transplanted them into an ischemia-reperfusion (I/R) injury model. CM was also injected into mice, and the histological changes, level of cell proliferation, loss of peritubular capillaries and anti-inflammatory and anti-apoptotic effects were examined at different time points.

**Results:**

The results showed that MSC infusion improved renal function and histological alterations, leading to significantly reduced mortality. MSC administration also promoted kidney microvasculature repair, attenuated kidney peritubular capillary loss, increased the proliferation of parenchymal cells and decreased CD68-positive macrophage infiltration and apoptotic cells. Although we determined that CM contained proangiogenic factors, including hepatocyte growth factor (HGF), vascular endothelial growth factor-A (VEGF-A) and insulin-like growth factor-1 (IGF-1), no favorable effects were observed during the course of repair.

**Conclusions:**

Our data show that MSC infusion promotes kidney repair in a variety of ways, including enhancement of the repair of peritubular capillaries and tubular epithelial cells and anti-inflammatory and anti-apoptotic effects. MSCs can secrete high levels of proangiogenic growth factors, but CM results in a nonsignificant improvement, indicating that MSCs play a role in kidney repair through paracrine rather than endocrine mechanisms. These results indicate that MSC infusion is a promising therapeutic strategy for promoting kidney repair after injury.

**Electronic supplementary material:**

The online version of this article (doi:10.1186/scrt489) contains supplementary material, which is available to authorized users.

## Introduction

Acute kidney injury (AKI) is one of the most important causes of mortality and morbidity worldwide. In clinical practice, kidney ischemia–reperfusion (I/R) is the most common cause of AKI. Limitations in the treatment have led to a search for better therapeutic options. Mesenchymal stem cell (MSC)-based therapy holds great promise for treating immune disorders and for regenerative medicine, and promising results have been reported for the application of different types of stem cells in the treatment of kidney failure [1–10]. Our previous studies have shown that hematopoietic stem cells are recruited to the kidney, attenuate peritubular capillary loss, promote tubular epithelial regeneration and prolong survival in diabetic mice [8].

Increasing studies have indicated that the beneficial effects of stem cells are primarily mediated via the paracrine/endocrine action of mediators rather than the direct differentiation and substitution of damaged cells [11–14], and many studies have shown that MSCs can secrete a wide range of growth factors and mediators that can suppress local immunologic reactions and inhibit fibrosis and apoptosis [2, 12, 15, 16]. According to these data, the direct injection of the supernatant from cultured MSCs may have beneficial effects on kidney repair.

In the present study, we cultured MSCs and harvested the supernatant as conditioned medium (CM). We then investigated the therapeutic potential of MSCs and CM administered 24 hours after kidney I/R injury. We observed that MSCs but not CM contributed to vascular regeneration, functional recovery, decreased macrophage infiltration and apoptotic cells and promoted survival.

## Materials and methods

### Animals

Male BALB/C mice (Harbin Medical University 2nd Affiliated Hospital Laboratories) were used at an age of 6 to 8 weeks and weighed between 20 and 25 g. All procedures involving animals were approved by the animal committee of Harbin Medical University.

### Animal model

The procedure for I/R injury of the kidney was modified from a method described previously [17]. In brief, on day 0 the kidneys of anesthetized male mice were exposed through surgical incisions in the flank, and at a core temperature of 36.8 to 37.3°C a nontraumatic microaneurysm clamp was placed across the renal artery and vein of either one or both kidneys. The kidneys were confirmed to be dusky and were then placed back into the retroperitoneum for 30 minutes (unilateral model) or 28 minutes (bilateral model). The clamps were removed and reperfusion of the kidneys was confirmed visually, and then the incision was closed. The CM was generated as follows: 2 × 10^6^ MSCs were cultured with 2 ml serum-free Dulbecco’s modified Eagle’s medium (DMEM; HyClone, Logan, UT, USA) for 48 hours, and the supernatant was subsequently separated from cells by filtering through a 0.22 μm filtration unit (Millipore, Bedford, MA, USA). To test the effect of MSCs and CM, mice subjected to unilateral I/R injury were divided into four groups. In the MSC group (*n* = 6/group) on day 1 after kidney injury, 200 μl MSCs (10^6^/ml) labeled with 5-chloromethylfluorescein diacetate (CMFDA) was infused intravenously through the tail vein. A total volume of 200 μl CM, DMEM or phosphate-buffered saline (PBS) was injected once per day from day 1. To evaluate renal function, mice with bilateral I/R kidney injury were randomly divided into four groups (*n* = 26/each group). These mice were injected with the same amounts of MSCs, CM, DMEM and PBS as the unilateral model. The plasma creatinine and blood urea nitrogen (BUN) levels were analyzed using plasma samples taken from the tail vein on days 1, 2, 3, 5 and 7 after injury.

### Isolation and expansion of MSCs

MSCs were isolated and cultured from the bone marrow of 6-week-old to 8-week-old male BALB/C mice using the method of Peister and colleagues [18]. Briefly, MSCs were generated by flushing the femur and tibia of anesthetized mice with PBS. The cell pellets were plated in culture dishes with high-glucose DMEM supplemented with 10% fetal bovine serum (HyClone) and 1% penicillin–streptomycin solution at 37°C and 5% carbon dioxide in air. The nonadherent cells were removed by changing the medium at 48 hours and every 72 hours thereafter. When the cells reached near confluence, they were removed from the dishes using 0.25% ethylenediamine tetraacetic acid–trypsin and passaged at a low density for four expansions. In this experiment, to track MSCs following systemic administration, the MSCs were adjusted to 10^6^/ml and labeled with 10 μM green fluorescent tracer CMFDA (Invitrogen, San Diego, CA, USA) for 30 minutes at 37°C. After further centrifugation, the cells were resuspended in PBS and kept on ice until infusion.

### Characterization of MSCs

MSCs were confirmed by the typical spindle-shaped appearance, by differentiation into osteocytes and adipocytes with specific differentiation media, and by fluorescence-activated cell sorting analysis using a BD FACS Calibur flow cytometer (BD Biosciences, San Diego, CA, USA) to assess the following markers: CD44, CD73, CD90, CD105, CD45, CD34 and CD11b. All antibodies and their respective isotype controls were purchased from BD Biosciences.

### Biochemical analysis and enzyme-linked immunosorbent assay

Approximately 50 μl blood samples were taken from the tail vein and centrifuged at 5,000 × *g* for 10 minutes at 4°C. The creatinine and the urea levels were measured using the kinetic Jaffe and enzymatic method. A total of 10^6^ MSCs from the fourth passage were plated on culture dishes in DMEM supplemented with 10% fetal bovine serum and 1% penicillin–streptomycin solution and were cultured for 24, 48 or 72 hours in an incubator. The supernatant was then collected and aliquots of 100 μl media were assayed for hepatocyte growth factor (HGF), vascular endothelial growth factor-A (VEGF-A) and insulin-like growth factor-1 (IGF-1) using an enzyme-linked immunosorbent assay according to the supplied protocols (Blue Gene, Shanghai, China). Control medium (DMEM plus 10% fetal bovine serum not cultured with MSCs) was also tested.

### Histology and immunostaining

Mice were perfused with ice-cold PBS, and the kidney tissues were fixed in periodate–lysine–paraformaldehyde fixative for 2 hours followed by 18% sucrose overnight. These tissues were then preserved in optimum cutting temperature compound (−80°C). The tissue used for light microscopy was fixed in 10% neutral-buffered formalin for 12 hours, transferred to 70% ethanol, processed to produce paraffin sections (3 μm) and stained with hematoxylin and eosin. Immunofluorescence labeling was performed on 4 μm cryosections. Mouse vasculature was labeled with rat-anti-mouse CD31 (1:100; eBioscience, San Diego, CA, USA). Cell proliferation was assessed using KI67 antigen labeling (1:100; Thermo, Ely, UK) and macrophage infiltration labeled with anti-CD68 (1:200; Abcam, Cambridge, UK). Terminal deoxynucleotidyl transferase-mediated dUTP nick end labeling (TUNEL) was carried out using an *in situ* cell death detection kit (Roche, Indianapolis, IN, USA) according to the manufacturer’s instructions. The number of these cells in the left kidney was counted from 10 different fields for each sample and averaged. Histological and immunofluorescent images were primarily from the cortical and outer medullary regions of the kidney. Peritubular capillary loss and tubular injury were evaluated by assessing anti-CD31-IgG TRITC-labeled kidney sections and hematoxylin and eosin-stained paraffin-embedded sections, respectively, using a blinded scoring method as previously reported [8]. In brief, images were captured by digital imaging (×200 magnification) sequentially over the entire sagittal section incorporating the cortex and outer medulla (10 images). Each image was divided into 252 squares by a grid. To calculate peritubular capillary loss, each square without a peritubular capillary resulted in a positive score, with the final score presented as a percent positive score. To assess tubular injury, each square with the presence of tubule injury (tubule flattening, necrosis, apoptosis or presence of casts) resulted in a positive score. The final score was the percentage of squares with a positive score, which was averaged for all images from the individual kidney. Confocal images were generated using an OLYMPUS FLUOVIEW FV1000 (Tokyo, Japan) confocal microscope.

### Statistical analysis

All data were presented as the mean ± standard deviation. The Kaplan–Meier test was used to analyze survival. The *t* test was used for group comparisons. Analyses were performed with SPSS software version 17 (SPSS Inc, Chicago, USA). *P* < 0.05 was considered significant in all statistical tests.

## Results

### Mesenchymal stem cell phenotype

**Figure 1:**
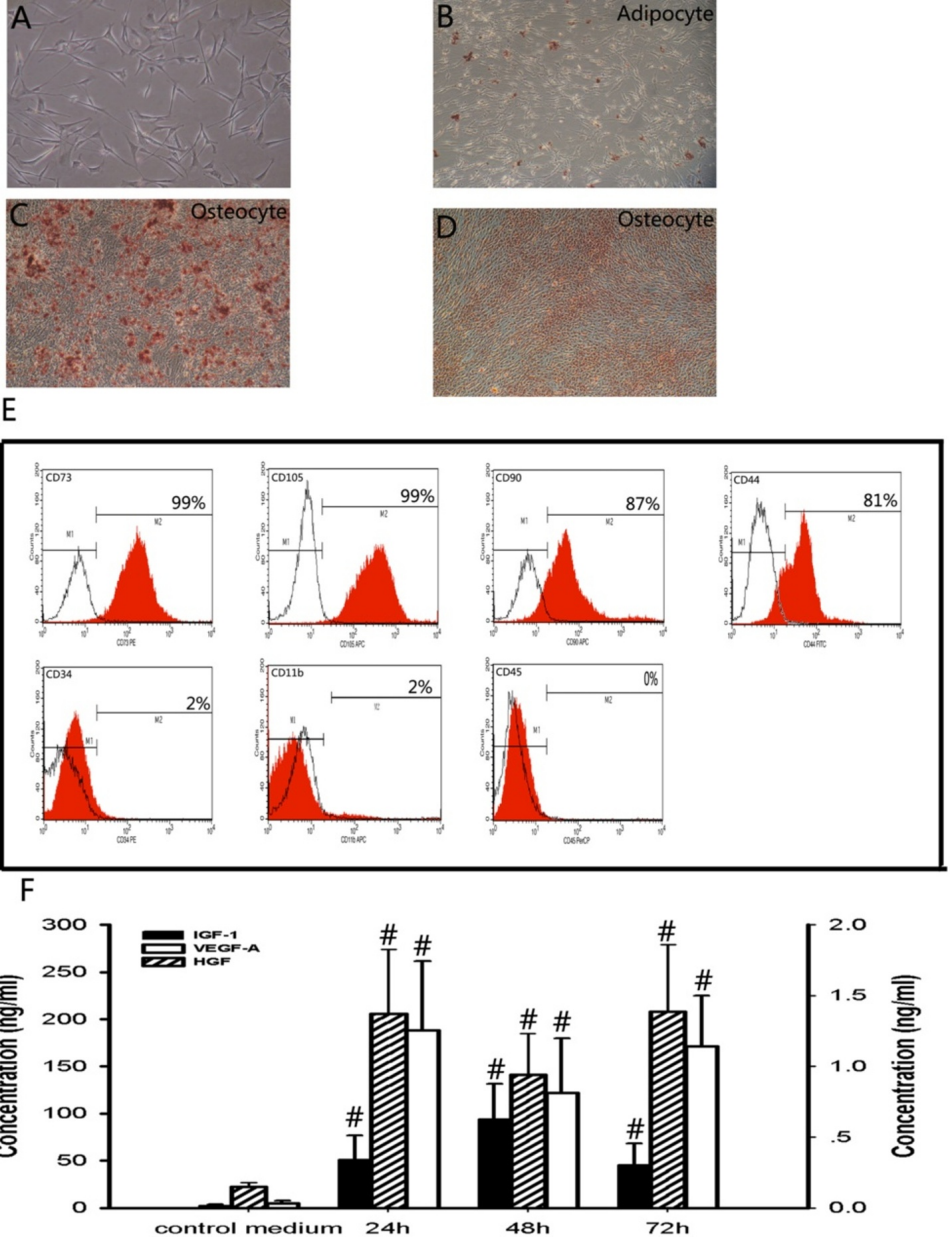
**Mesenchymal stem cell phenotype and levels of the growth factors HGF, VEGF-A and IGF-1 in conditioned medium at 24, 48 and 72 hours. (A)** Light microscopy revealed that the mesenchymal stem cells (MSCs) were spindle-shaped. **(B)** Oil Red O staining determined differentiation to adipocytes. **(C)** Alizarin Red S was used to show differentiation to osteocytes. **(D)** Alkaline phosphatase staining also confirmed differentiation to osteocytes. Magnification, ×100. **(E)** Representative flow cytometry graphs of surface markers. The cells were positive for CD44, CD73, CD90 and CD105 and were negative for CD45, CD34 and CD11b. Red areas, tested antibodies; white areas, isotype controls. **(F)** Hepatocyte growth factor (HGF), vascular endothelial growth factor-A (VEGF-A) and insulin-like growth factor-1 (IGF-1) levels in conditioned medium and control medium (*n* = 8). IGF-1 and VEGF-A shown on the left and HGF on the right of the *y* axis. Data presented as mean ± standard deviation. ^*#*^ *P* < 0.01 versus control medium.

MSCs were generated according to standard procedures, and the nonadherent cells were removed by a medium change. MSCs were confirmed using light microscopy to verify the typical spindle-shaped morphology (Figure 1A). The identity of these cells was determined by differentiation to osteocyte and adipocyte lineages and surface marker analysis, which showed that the cells were positive for CD44, CD73, CD90 and CD105 and were negative for CD45, CD11b and CD34 (Figure 1B,C,D,E). Only MSCs that met these criteria were used in subsequent experiments.

### Conditioned medium contains the growth factors HGF, VEGF-A and IGF-1

First, we examined the number of MSCs cultured with serum-free DMEM for 24, 48 and 72 hours. We found by light microscopy that there were no significant differences at different time points (see Figure S1A,B,C,D in Additional file 1). Also, no significant differences of cell viability and cell death rates at different time points were found by MTT and trypan blue staining (see Figure S1E,F in Additional file 1). Second, to investigate the mechanisms by which MSCs repair AKI, we determined whether the growth factors HGF, VEGF-A and IGF-1 were present in CM. We found that the MSCs produced high levels of the proangiogenic growth factors and that there were no significant differences at different time points, but that results were significantly higher than with control medium (Figure 1F). Based on these data, we hypothesized that CM, when used alone, may promote kidney repair after I/R injury.

### Chloromethylfluorescein diacetate-labeled MSCs are recruited to the kidney during repair after I/R injury

**Figure 2:**
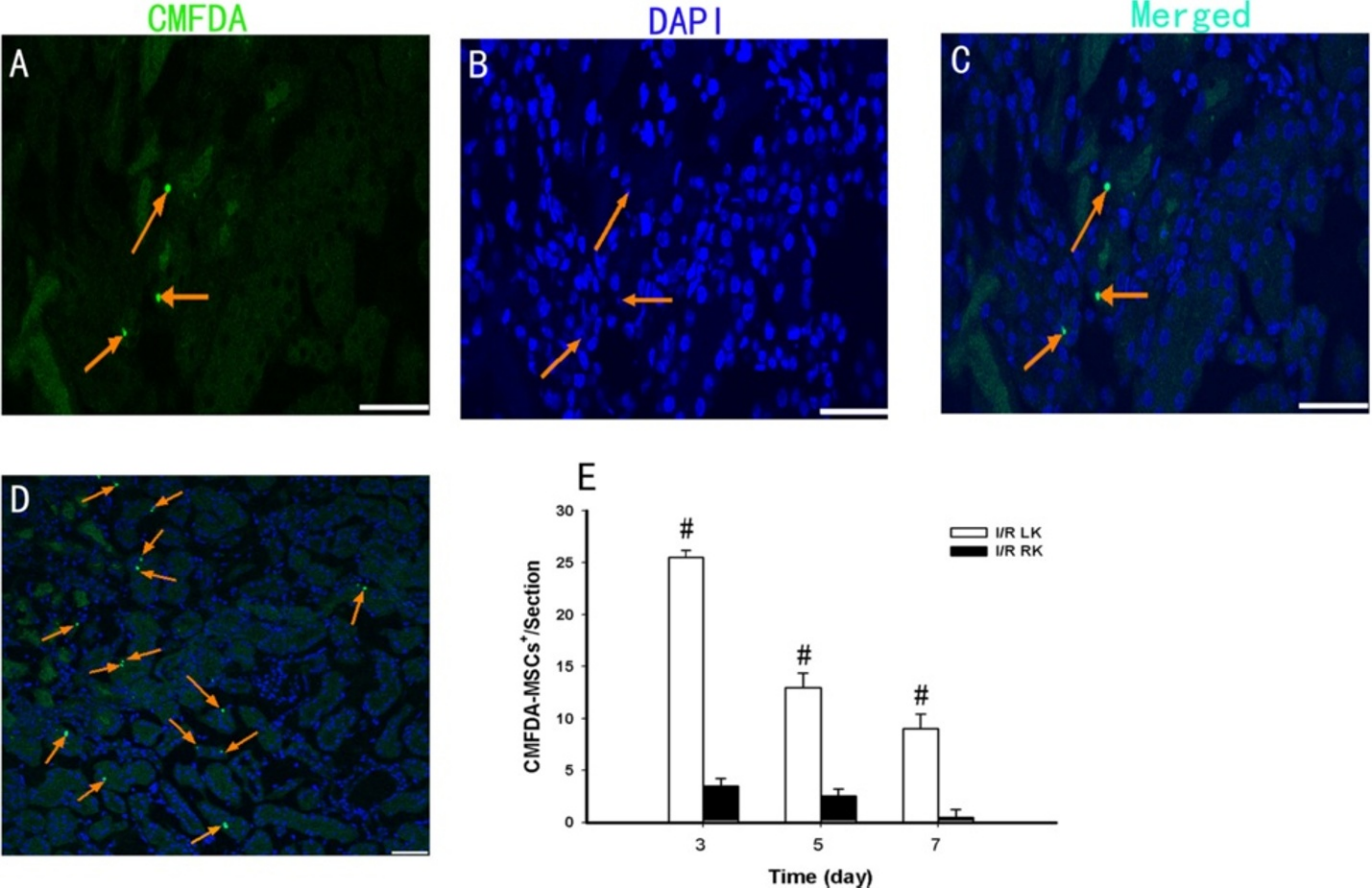
**Mesenchymal stem cells are recruited to the injured kidney.** 5-Chloromethylfluorescein diacetate (CMFDA)-labeled mesenchymal stem cells (MSCs) with **(A)** the individual CMFDA, **(B)** 4′,6-diamidino-2-phenylindole (DAPI), and **(C)** merged channels on day 3 after injury. **(D)** CMFDA-labeled MSCs were present in the injured kidney (arrowheads) on day 3 post ischemia–reperfusion (I/R) injury. **(E)** Number of MSCs observed in injured and control kidneys over time after I/R injury (*n* = 6). Data presented as mean ± standard deviation. ^#^ *P* < 0.01 versus the control kidney (bars, 50 μm). LK, left kidney; RK, right kidney.

To track MSCs following systemic administration, cells were labeled with the green fluorescent tracer CMFDA. To study the effects of MSCs on kidney repair, we initially determined whether they could be recruited to the injured kidneys. We infused CMFDA-labeled MSCs via the tail vein on day 1 after unilateral I/R injury and examined the kidneys on days 3, 5 and 7 after I/R injury by confocal microscopy. We observed that MSCs were recruited to the kidneys, most of them localized to the cortical and medullary tubular tissue of injured kidneys, especially in the outer medulla where the proximal tubules are located (Figure 2D), although a few were found in contralateral kidneys, indicating that MSC recruitment to the injured kidneys was the result of a specific process and that the number of recruited MSCs decreased over time (Figure 2E). Enlarged views showed that every CMFDA-labeled MSC presented a nucleus counterstained with 4′,6-diamidino-2-phenylindole (Figure 2A,B,C). We found that many MSCs could be observed in the lungs and spleen, but no cells were found in the heart (data not shown), consistent with our previous study [8].

### Mesenchymal stem cells, not conditioned medium, improve renal function and enhance survival

**Figure 3:**
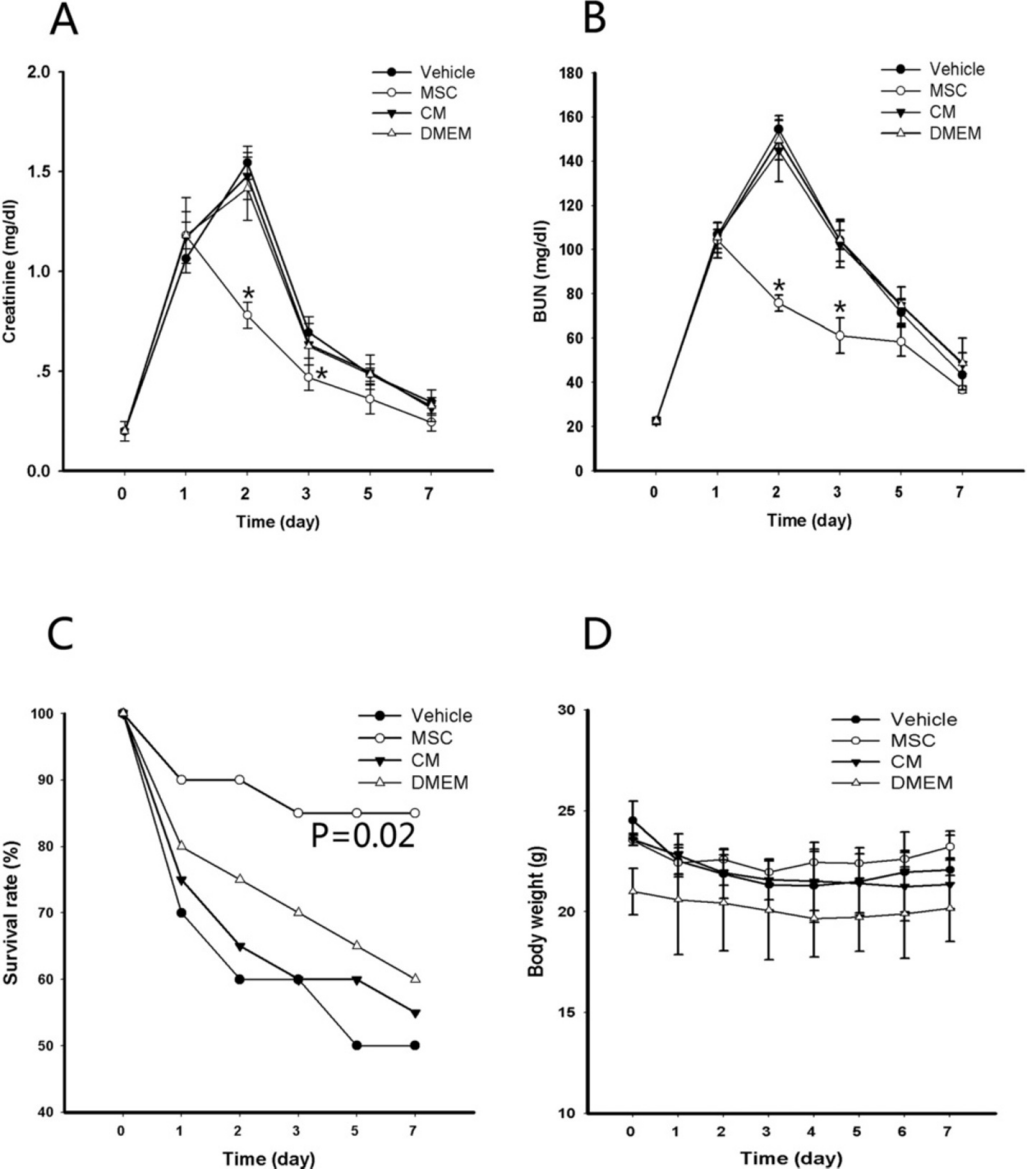
**Administration of mesenchymal stem cells, not conditioned medium, improves renal function and reduces mortality. (A)** Creatinine and **(B)** blood urea nitrogen (BUN) levels at each time point for bilateral ischemia–reperfusion (I/R) model mice treated with phosphate-buffered saline, mesenchymal stem cells (MSCs), conditioned medium (CM) or Dulbecco’s modified Eagle’s medium (DMEM) (*n* = 26/each group). Data presented as mean ± standard deviation. **(C)** Survival curves at each time point for mice subjected to bilateral I/R injury followed by intravenous injection with MSCs, vehicle, DMEM or CM (*n* = 20/each group, *P* = 0.02 MSC group vs. vehicle group). **(D)** Body weight curves during the study period for the MSC group, DMEM group, CM group and vehicle group. *P < 0.05 versus vehicle group.

We subjected the mice to bilateral I/R injury for 28 minutes (day 0), followed by intravenous infusion of CM or MSCs on day 1. As shown in Figure 3A,B, the renal function level was assessed in sham surgery mice on day 0 (creatinine, 0.19 ± 0.049 mg/dl; BUN, 22.54 ± 0.68 mg/dl). Bilateral kidney I/R injury resulted in significant increases in creatinine and BUN levels on day 1 (creatinine, 1.16 ± 0.05 mg/dl; BUN, 106.87 ± 2.33 mg/dl), and these levels peaked on day 2 (creatinine, 1.54 ± 0.083 mg/dl; BUN, 154.41 ± 6.14 mg/dl) and declined on day 3 but did not return to normal levels by day 7. There were marked decreases in creatinine and BUN levels on days 2 and 3 in the MSC group compared with the vehicle group. However, CM and DMEM did not result in any improvement in renal function, and there were no significant differences between the vehicle group and either the CM group or the DMEM group. To evaluate the survival rate, the mice were subjected to bilateral I/R injury and treated with MSCs, vehicle, CM or DMEM. Only 50% of the mice survived to day 7 in the vehicle group, whereas 85% of the mice that received MSCs survived (*P* = 0.02). As shown in Figure 3C, there were no significant improvements in the CM or DMEM groups (*P* = 0.748 and *P* = 0.493, respectively) compared with the vehicle group. In this experiment, we also assessed the body weight of the mice in the four groups. There was a marked and significant decrease at 24 hours after bilateral I/R injury, and in the MSC group there was a slight and persistent enhancement on day 2. These mice almost reached their presurgery body weight by the end of the study period, but in the other three groups the body weight remained lower than that pre surgery, although there were no differences among the four groups (Figure 3D). These findings indicate that MSCs can promote renal function, enhance survival and increase body weight. However, no significant differences were observed between the vehicle group and either the CM group or the DMEM group.

### Mesenchymal stem cell administration ameliorates histological alterations and attenuates kidney peritubular capillary loss, but conditioned medium has no beneficial effects

**Figure 4:**
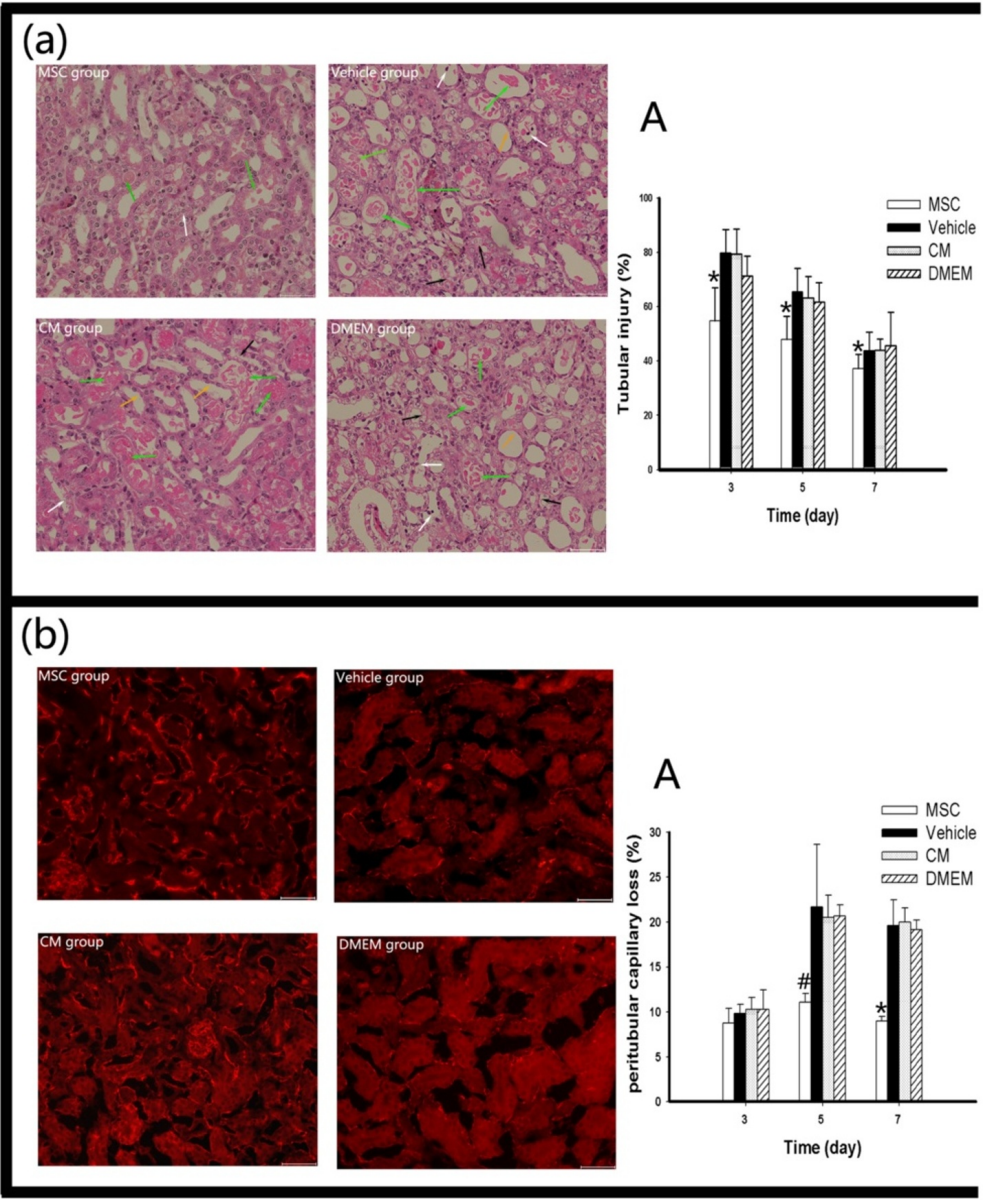
**Mesenchymal stem cell administration prevents tubular injury and attenuates kidney peritubular capillary loss, but conditioned medium has no effect. (a)** Representative light microscopy images of hematoxylin and eosin-stained kidney sections on day 3 in the mesenchymal stem cell (MSC) group, vehicle group, conditioned medium (CM) group or Dulbecco’s modified Eagle’s medium (DMEM) group. White arrow, cell nuclei defluxion; black arrow, cell nuclei dissolution and absorption; yellow arrow, tubular atrophy; green arrow, cellular debris and proteinuria cast. **(A)** Tubular injury index on days 3, 5 and 7 for different groups. **(b)** Immunofluorescent images of CD31-labeled peritubular capillaries on day 7 after ischemia–reperfusion injury in mice that received MSCs, vehicle, CM or DMEM. **(A)** Peritubular capillary loss index for mice in the four groups at different time points. Data presented as mean ± standard deviation. *P < 0.05 versus the vehicle group, ^#^ *P* < 0.01 versus the vehicle group (bars, 250 μm).

Tubular injury was examined on days 3, 5 and 7 after I/R injury, and the sections were stained with hematoxylin and eosin. The light microscopy findings showed that MSC administration significantly attenuated tubular injury compared with vehicle administration. We did not observe any significant difference between the CM and DMEM groups. The tubular injury index of each for the four groups during the repair phase of this model was evaluated (Figure 4a).Because peritubular capillaries play a central role in kidney function, we also analyzed kidney sections for the loss of peritubular capillaries during repair. The analysis of mCD31-labeled peritubular capillaries by morphometry revealed that MSC treatment prevented peritubular capillary loss during the repair phase through day 7 after I/R injury, and the results were significant on days 5 and 7. In contrast, with CM and DMEM treatment we did not observe any beneficial effect on vasculature repair during the repair phase of AKI. The percentage of peritubular capillary loss in each of the four groups on days 3, 5 and 7 was scored (Figure 4b).

### Mesenchymal stem cells, not conditioned medium, promote the proliferation of parenchymal cells and significantly decrease CD68-positive macrophage infiltration and apoptotic cells

**Figure 5:**
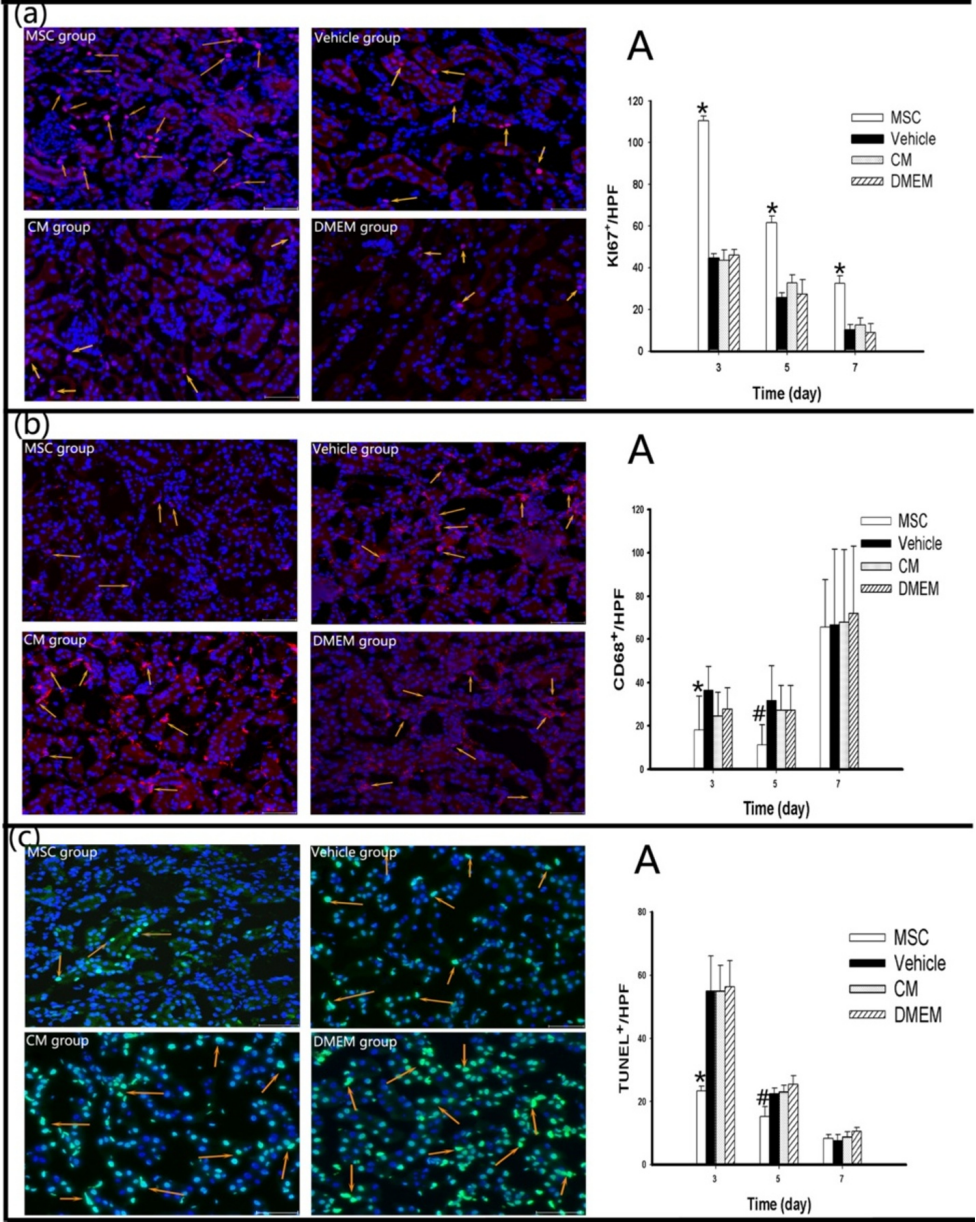
**Mesenchymal stem cell treatment promotes the proliferation of parenchymal cells and attenuates CD68** ^**+**^ **cell infiltration and TUNEL-positive cells, but conditioned medium administration has no beneficial effect. (a)** Images of the immunofluorescent staining of KI67^+^ cells (yellow arrows) in the injured kidneys of mice on day 3 after ischemia–reperfusion (I/R) injury in the mesenchymal stem cell (MSC) group, vehicle group, conditioned medium (CM) group and Dulbecco’s modified Eagle’s medium (DMEM) group. **(A)** Number of KI67^+^ cells in the four groups on days 3, 5 and 7 after I/R injury. **(b)** Representative confocal images of the immunofluorescent staining of CD68^+^ cells (yellow arrow) in the injured kidneys of mice on day 3 after I/R injury in the MSC group, vehicle group, CM group and DMEM group. **(A)** Number of CD68^+^ cells in the four groups on days 3, 5 and 7 after I/R injury. **(c)** Effect of different treatments on I/R-induced tubular apoptosis on day 3 after injury. Kidneys from mice treated with MSCs, vehicle, CM and DMEM were stained using the terminal deoxynucleotidyl transferase-mediated dUTP nick end labeling (TUNEL) assay. **(A)** Number of TUNEL^+^ cells in the four groups at different time points. Data presented as mean ± standard deviation. *P < 0.05 versus the vehicle group, ^#^ *P* < 0.01 versus the vehicle group (bars, 250 μm).

In this study we investigated whether MSCs and CM promoted cell proliferation. The cell cycle marker KI67 was used to evaluate proliferation. Our studies revealed that MSC infusion led to a marked increase in the number of parenchymal cells in the cell cycle after injury. However, we did not observe any significant difference between the vehicle group and the CM group or the DMEM group. The quantification of KI67^+^ cells at different time points in four groups is shown in Figure 5a.I/R animals exhibited prominent infiltration of CD68-positive macrophages in the tubulointerstitial compartment of the renal cortex and outer medulla in kidneys at 3 days post AKI (Figure 5b), consistent with the acute inflammatory response following I/R injury. MSC infusion dramatically reduced macrophage infiltration into the post-ischemic kidney, especially on day 5. In contrast, there was no significant decrease in the CM group or the DMEM group compared with the vehicle group.

Apoptosis is a critical pathophysiological event in AKI. We therefore evaluated TUNEL staining of the kidneys after I/R. As shown in Figure 5c, there were many TUNEL-positive cells in renal tubular at 3 days post I/R injury. The number of TUNEL-positive cells was significantly decreased with time, and MSC administration significantly decreased the number of TUNEL-positive cells on day 3, especially on day 5, but CM and DMEM administration did not significantly reduce tubular cell apoptosis. Light and immunofluorescence images from animals with a sham surgery on days 3, 5 and 7 are shown in Additional file 2.

### No effective results were observed after administration of a high dose of conditioned medium to mice

Considering that most of the mediators in CM would probably have a short half-life and be present at low concentrations, we wondered whether consecutive and high-dose injections of CM are necessary for protection against kidney injury. Hence, we performed the latter experiment by daily administration of 500 μl CM (*n* = 6) for 7 days starting from day 1 after I/R injury, where the mice were killed on days 3, 5, and 7. Similar to our previous result, there were no significant differences in the examined parameters in mice receiving CM (see Additional file 3). This experiment therefore revealed that even consecutive and high-dose administration of CM was not effective in ameliorating I/R injury.

## Discussion

AKI continues to result in high morbidity and mortality, particularly in patients admitted to the ICU [5, 19–21]. In addition, emerging evidence indicates that AKI in humans is closely associated with chronic kidney disease if the repair process is maladaptive [22, 23]. However, the therapeutic options are limited.

Bone marrow stem cells are an attractive therapy to promote renal tissue regeneration due to their pluripotency and ease of isolation. Using these cells also avoids the ethical ambiguities of using embryonic stem cells [4, 15, 24, 25]. Our previous studies also demonstrated that hematopoietic stem cells recruited to injured kidneys generate high levels of proangiogenic cytokines, including VEGF-A [8]. This result increased our interest in determining whether CM had beneficial effects on kidney repair.

In the present study, we obtained MSCs using typical methods and cultured these cells for four passages before use in our experiments. Light microscopy showed that these cells had typical spindle-shaped morphology and were well labeled with CMFDA. Additionally, we demonstrated that MSCs that were systemically infused 24 hours after kidney injury were selectively recruited to injured kidneys. This recruitment was associated with enhanced repair of the microvasculature and tubules, improved kidney function, increased survival, promoted the proliferation of parenchymal cells, and decreased CD68-positive macrophage infiltration and apoptotic cells. In contrast, systemic CM treatment did not have any significantly beneficial effects, even though the CM contained high levels of proangiogenic cytokines, including HGF, VEGF-A and IGF-1.

Acute ischemic injury in the kidneys primarily results in proximal tubular damage [6, 26, 27]. However, data derived from several severe AKI models and the long-term effects of ischemic injury demonstrate that capillary loss typically precedes the development of prominent renal fibrosis, the loss of capillary density and blood flow may result in poor delivery of oxygen and nutrients to the damaged area, and neoangiogenesis may be a central process in the preservation of the vascular structure and the restoration of organ function [28–31]. In this study, we demonstrated that there was a marked loss of peritubular capillaries in the injured kidneys, and that the intravenous infusion of MSCs attenuated the loss of peritubular capillaries and tubular injury and promoted cell proliferation in the kidney. These effects were associated with both the rapid recovery of kidney function and the enhanced survival of the mice.

The critical property of stem cells is that they are able to generate many or all differentiated cell types [32, 33]. Initial studies reported that bone-marrow derived stem cells can differentiate into endothelial and mesangial cells in animal models [34–36], but the number of differentiated cells was small. Recently, it was found that MSCs can produce many growth factors, suggesting that a paracrine/endocrine effect might contribute to renal protection [2, 4, 12]. Gharaibeh and colleagues have shown that the terminal differentiation capacity of implanted stem cells is not the major determinant of the cells’ regenerative potential and that the paracrine effect imparted by the transplanted cells plays a greater role in the regeneration process [37]. Zarjou and colleagues have further shown that heme oxygenase-1 enhances secretion of stromal cell-derived factor-1, VEGF-A and HGF by MSCs [38]. Many findings support a protective effect mediated in an endocrine manner, which, if true, would mean that injection of the cells themselves would not be required, and the factors that these cells secrete could be effective. The effect of CM, however, remains controversial for the moment [12, 39]. In this study we also determined the levels of HGF, VEGF-A and IGF-1, and the data showed that CM contained these factors, which have renoprotective effects after AKI. Based on these results, we hypothesized that administering the CM would protect against kidney failure, making it unnecessary to transplant stem cells and thus avoiding the risks of tumorigenesis and immunologic reactions. However, we did not observe any favorable effects in the CM group on renal function, histological alterations or cell proliferation and anti-inflammatory and anti-apoptotic effects, even though we increased the dose and repeated consecutive administration of CM. There are several possible explanations for these findings. First, the AKI injury models were induced by different methods, and we believe that the outcomes should be compared within a unique and identical model and cannot be meaningfully transposed from one model to another. Second, the microenvironment has very important effects on the production of growth factors by MSCs. Different microenvironments can stimulate stem cells to release different types and concentrations of cytokines. MSCs might secrete another set of mediators in the culture system [12]. If we want stem cells to have the same effects *in vitro* and *in vivo*, we must mimic the injury microenvironment in the culture system. In the I/R model, the loss of blood flow results in hypoxia in the tissue, and the bone marrow is also hypoxic [40, 41]. We therefore believe that the MSCs should be exposed to hypoxic conditions to mimic the *in vivo* environment. Some authors have performed these types of experiments [42–44]. Third, the timing of therapeutic cell delivery may be critical. Cellular populations within wounds change depending on the phases of the repair process. This change means that therapeutic cells will encounter different microenvironments at each stage of the repair process [45].

In contrast with our data, Bi and colleagues reported that administration of MSC CM was very potent in ameliorating cisplatin-induced kidney failure [12]. Comparing these two studies, there are some differences. First, the medium was harvested after 96 hours as CM but in our study was harvested after 48 hours. Second, Bi and colleagues infused 1000 μl CM twice per day for 6 days by intraperitoneal injection, and we injected 200 μl or 500 μl CM intravenously through the tail vein once per day for 7 days. Third, they gave an intraperitoneal injection of cisplatin to induce acute tubular injury, but we placed a nontraumatic microaneurysm clamp across the renal artery and vein to induce kidney I/R injury. Fourth, different mouse strains were used in these two studies (C57BI/6 compared with BALB/C). We consider that these differences account for the discrepancies in the findings at least in part. We believe the that therapeutic strategy for treatment of kidney disease with CM remains an open question, and further studies with different designs, animal models and evaluation methods are certainly required.

## Conclusions

We demonstrate that systematically administered MSCs promote rapid kidney repair and reduce mortality. Our data supporting the fact that the beneficial effect seen with MSCs is probably due to the stem cells’ multipotent capacity include increased secretion of paracrine factors, improved angiogenic and anti-inflammatory activities and anti-apoptotic effects. The results of this study indicate that the MSC infusion is a promising therapeutic strategy for AKI. In the present study, we do not detect any beneficial role of CM in our animal model, indicating that MSCs play central roles in kidney repair through paracrine rather than endocrine mechanisms. We believe that considerable work with different designs and animals is still required.

## Electronic supplementary material

Additional file 1: **is Figure S1 showing light microscopy images of MSCs at 0, 24, 48 and 72 hours of culture with fetal bovine serum (FBS)-free DMEM, examined by MTT and trypan blue staining.** (A to D) Light microscopy of MSCs at 0, 24, 48 and 72 hours of culture with FBS-free DMEM. Graph showing MTT cell viability assay (E) and trypan blue staining (F) cultured with FBS-free DMEM at different time points, ^#^ *P* < 0.01 versus the control medium (DMEM not cultured with MSCs). (JPEG 3 MB)

Additional file 2: **is Figure S2 showing images from animals for histological and immunofluorescent assessments on days 3, 5 and 7 after sham surgery.** There were no significant differences between different time points for histological evaluation (a), peritubular capillary loss (b), KI67^+^ cells (c), CD68^+^ macrophages (d) and apoptotic cells (e). (JPEG 5 MB)

Additional file 3: **is Figure S3 showing that no effective results were observed after administration of 500 μl CM to mice.** There were no significant differences between the CM and vehicle groups when examined for histological alterations (a), capillary density (b), proliferation of parenchymal cells (c), macrophage infiltration (d) and TUNEL apoptotic cells (e). (JPEG 4 MB)

## Abbreviations

AKI: acute kidney injury
BUN: blood urea nitrogen
CM: conditioned medium
CMFDA: 5-chloromethylfluorescein diacetate
DMEM: Dulbecco’s modified Eagle’s medium
HGF: hepatocyte growth factor
I/R: ischemia–reperfusion
IGF-1: insulin-like growth factor-1
MSC: mesenchymal stem cell
PBS: phosphate-buffered saline
TUNEL: terminal deoxynucleotidyl transferase-mediated dUTP nick end labeling
VEGF-A: vascular endothelial growth factor-A.
