# Supplementary figures and images for: Mesenchymal stem cells, not conditioned medium, contribute to kidney repair after ischemia-reperfusion injury
Source: Stem Cell Res Ther. 2014 Aug 21;5(4):101. doi: 10.1186/scrt489 (PMC4159523; doi:10.1186/scrt489)

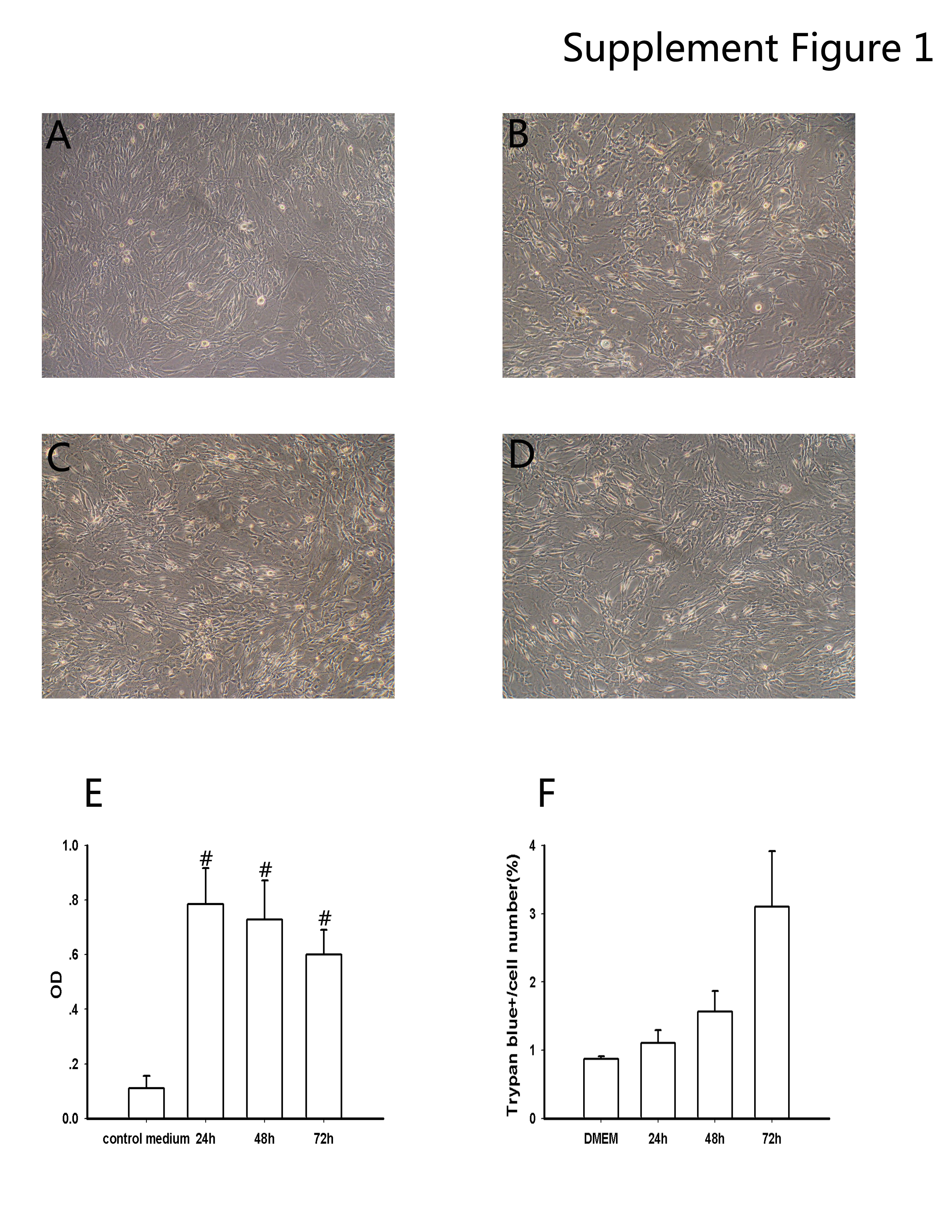

Supplement: Supplementary file 1 — Additional file 1: is Figure S1 showing light microscopy images of MSCs at 0, 24, 48 and 72 hours of culture with fetal bovine serum (FBS)-free DMEM, examined by MTT and trypan blue staining. (A to D) Light microscopy of MSCs at 0, 24, 48 and 72 hours of culture with FBS-free DMEM. Graph showing MTT cell viability assay (E) and trypan blue staining (F) cultured with FBS-free DMEM at different time points, # P < 0.01 versus the control medium (DMEM not cultured with MSCs). (JPEG 3 MB) [file 13287_2013_389_MOESM1_ESM.jpeg]

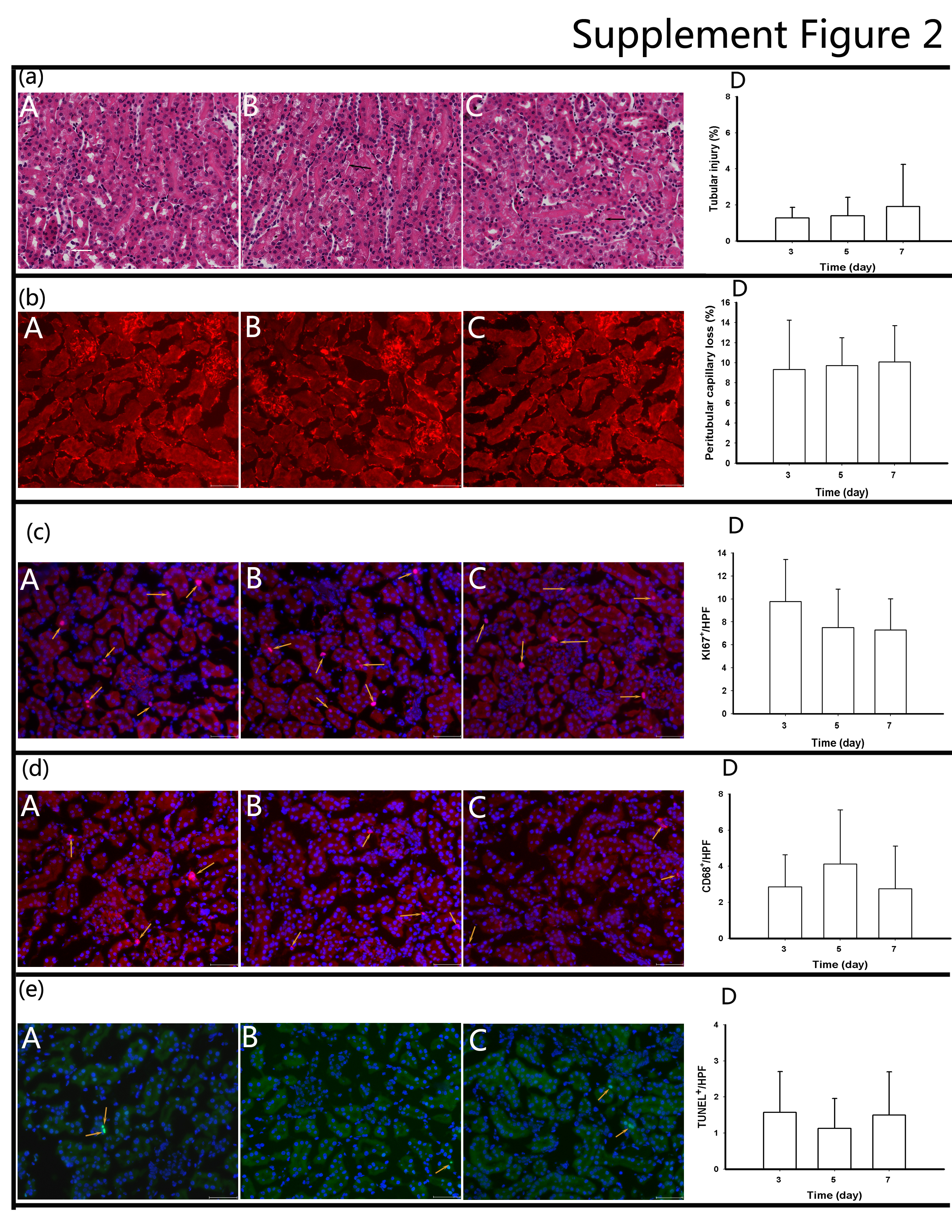

Supplement: Supplementary file 2 — Additional file 2: is Figure S2 showing images from animals for histological and immunofluorescent assessments on days 3, 5 and 7 after sham surgery. There were no significant differences between different time points for histological evaluation (a), peritubular capillary loss (b), KI67+ cells (c), CD68+ macrophages (d) and apoptotic cells (e). (JPEG 5 MB) [file 13287_2013_389_MOESM2_ESM.jpeg]

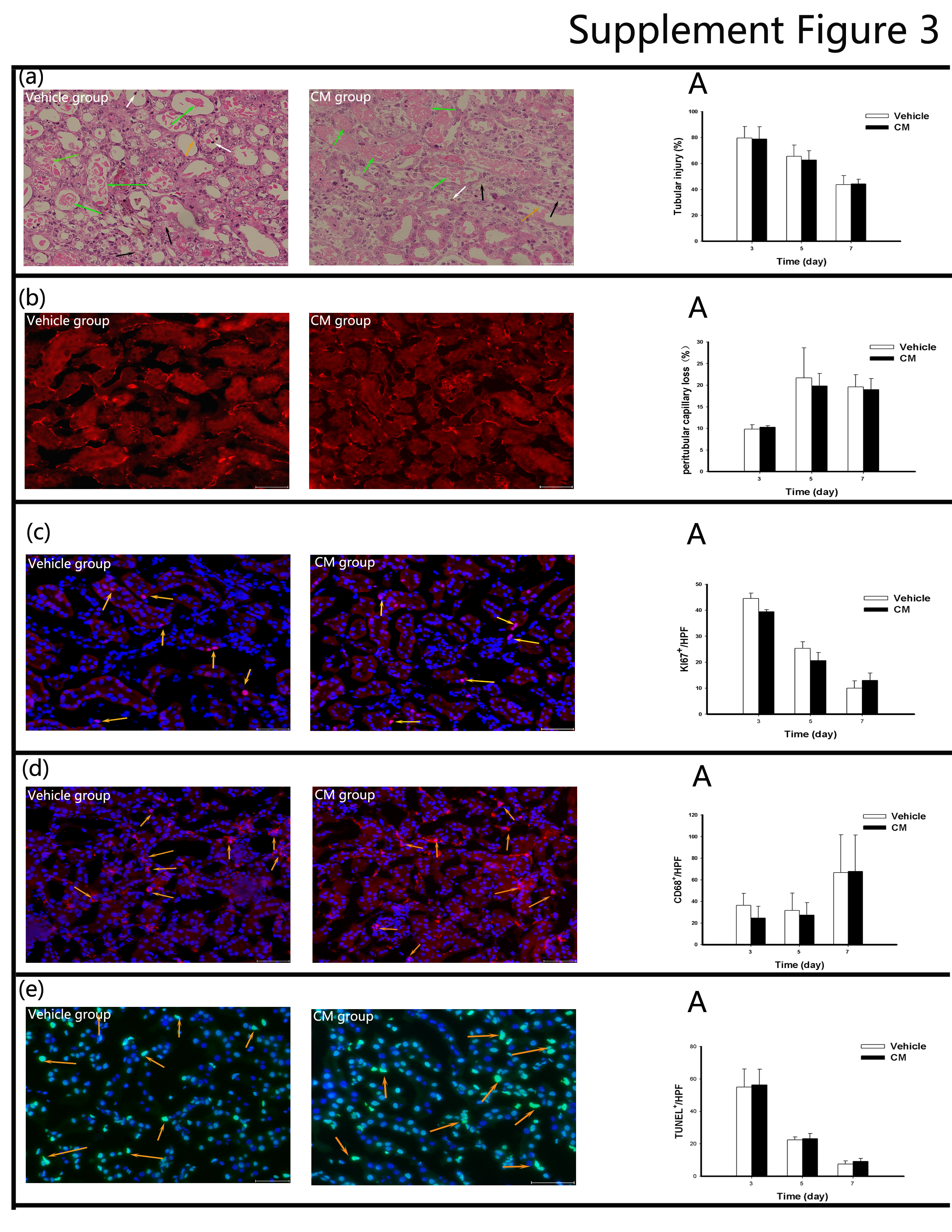

Supplement: Supplementary file 3 — Additional file 3: is Figure S3 showing that no effective results were observed after administration of 500 μl CM to mice. There were no significant differences between the CM and vehicle groups when examined for histological alterations (a), capillary density (b), proliferation of parenchymal cells (c), macrophage infiltration (d) and TUNEL apoptotic cells (e). (JPEG 4 MB) [file 13287_2013_389_MOESM3_ESM.jpeg]
